# Vinculin expression in MC3T3-E1 cells in response to mechanical stimulus

**DOI:** 10.1016/j.dib.2015.11.052

**Published:** 2015-12-05

**Authors:** J.J. Cora-Cruz, N. Diffoot-Carlo, P.A. Sundaram

**Affiliations:** a Department of Mechanical Engineering, University of Puerto Rico: Mayagüez Campus, Mayagüez, PR 00680, USA; b Department of Biology, University of Puerto Rico: Mayagüez Campus, Mayagüez, PR 00680, USA

**Keywords:** MC3T3-E1 Cells, Mechanical Stimulus, Focal Adhesions, Vinculin

## Abstract

Loading frequency is known to influence the expression of the focal adhesions of the adherent cells. A small cyclical tensile force was transmitted to mouse pre-osteoblast MC3T3-E1 cells through PDMS substrates of varying stiffness. Changes in cell behavior with respect to proliferation and characteristics of focal adhesions were quantified through immunofluorescence labeling of vinculin. Amount of inactive vinculin was higher on substrates subjected to cyclic stimulation when compared with the results of the static substrates, whereas the number and area of focal adhesion points underwent a reduction. Inactive vinculin appears as a cloud in the cytoplasm in the vicinity of the nucleus.

**Specifications Table**

**Table:** 

*Subject area*	*Biology*
*More specific subject area*	*Vinculin expression in MC3T3-E1 cells*
*Type of data*	*Table, image and text file*
*How data was acquired*	*Customized bioreactor, Scanning Laser Confocal Microscope*
*Data format*	
*Experimental factors*	*None*
*Experimental features*	*MC3T3-E1 cells were subjected to mechanical stimulus via PDMS substrates on which they were seeded. After static and cyclic stimulus, cells were viewed in a confocal microscope after appropriate immunofluorescent labeling for vinculin.*
*Data source location*	*Not applicable*
*Data accessibility*	*Data is with this article*

## 1 Value of the data

• Vinculin expression appears to increase under cyclic stimulus
• Fewer and smaller focal adhesion points for cyclic stimulus vis-à-vis static loading of substrate
• Vinculin appears as a cloud in the cytoplasm in the vicinity of the nucleus

## 2 Data

The characteristics of vinculin expression at focal adhesion points and in the cytoplasm are described through the use of a table and confocal microscope images.

## 3 Experimental design, materials and methods

### 3.1 Cell line

The preosteoblast MC3T3-E1 Subclone 4 cell line (ATCC, CRL-2593) was selected since it exhibits a high degree of differentiation and collagen production when grown in the presence of ascorbic acid. The MC3T3-E1 cell line is also a good model for understanding extracellular matrix signaling and hence tissue remodeling. It behaves similar to primary calvarial osteoblasts, which is ideal for modeling bone cells. The medium is composed of 90% Alpha Minimum Essential Medium (α-MEM_//_cellgro, 50-012-PB), 10% Fetal Bovine Serum (FBS_//_Thermo Scientific, SH30071.03) and antibiotic (Penicillin-Streptomycin Solution_//_cellgro, 30-002-CI). Ascorbic acid and Fungizone were only added to the medium to be used for experimentation. Cells were cultured at 37 °C and 5% CO_2_.

### 3.2 PDMS

Sylgard 184 Polydimethylsiloxane (PDMS) polymers were fabricated at 5:1, 10:1 and 15:1 base-to-crosslinker ratios. The appropriate ratios were obtained by proportionally adding and mixing the amount of volume required of each component. The mixture was then transferred to a centrifuge tube and centrifuged for 5 min at 4000 rpm in order to degas the mixtures. Subsequently they were slowly transferred to a mold until the desired substrate thickness was reached (0.5 mm). They were left at 60 °C for five hours and thereafter stored at room temperature. The PDMS film was then removed from the mold and cut to the desired dimensions. Three different PDMS substrates with varying stiffness based on the base-to-crosslinker ratio were prepared. Substrates will be referred to by their respective base component of base-to-crosslinker ratio henceforth. For example, a PDMS substrate at a 5:1 base-to-crosslinker ratio will be labeled as PDMS 5. The substrates were then washed with 95% ethanol for 30 min and subsequently left in fresh ethanol for two hours, following which they were submerged in phosphate buffer solution (PBS) 1X for an hour to remove any trace of ethanol that might have not evaporated. PDMS substrates were prepared with a thickness of 0.5 mm and cut into 65 mm×5 mm size samples. Each sample was then coated with fibronectin and placed in the bioreactor to apply the mechanical stimulus.

### 3.3 Fibronectin coating

Fibronectin extracted from bovine plasma (Sigma-Aldrich; St. Louis, Missouri | F1141) was diluted in PBS 1X to form a 5 µg/mL stock solution; it was stored at 4 °C as indicated by the manufacturer. The PDMS substrates were submerged in the coating solution for 45 min at room temperature before the removal of the solution. The substrates were then left an additional hour at room temperature to dry the remaining solution in air. Coating of the substrates was carried out under sterile conditions.

### 3.4 Mechanical stimulation

A chamber with three PDMS substrates (PDMS 5, PDMS 10, PDMS 15) of varying stiffness was mounted into the bioreactor and a strain of 1% at 0.05 Hz for 5 min intervals (*5* min *of loading followed by a no-load period of 5* min) was applied for four days. During experimentation, a second chamber (referred to as static chamber hereafter) with identical conditions except, that no load was applied, was used as a control. The static chamber does not have an intended load, although a minimal strain corresponding to the negligible tension required to maintain the substrates without flaccidity within the chamber was applied. MC3T3-E1 cells (45,000) were seeded in each of the chamber׳s wells. After the experimentation, the samples were prepared for immunofluorescence labeling and subsequently imaged through the use of confocal microscopy.

### 3.5 Immunofluorescence labeling

Samples were removed from their respective culture chambers after being cultivated for a period of 4 days (12 h seeding; 4 days experimentation). Immunofluorescence labeling of vinculin was performed. Two 5×10 mm^2^ pieces were carefully extracted from each sample, washed three times in PBS 1X and fixed for 20 min at room temperature through the use of 4% formaldehyde. They were then washed twice in washing buffer (PBS 1X containing 0.05% Tween20) to remove any remaining debris. Permeabilization was performed at room temperature using PBS 1X containing 0.15% Triton X-100 for 5 min, washed twice with washing buffer and left for 1 h in blocking solution (PBS 1X containing 1% BSA).

The specific primary antibody was diluted in blocking solution and left at room temperature for 1 h. Vinculin Monoclonal Antibody, purified clone 7F9 (EMD Millipore; Billerica, Massachusetts | 90227) was used to label the vinculin. The samples were then washed three times with washing buffer for 10 intervals. The secondary antibody, Alexa Fluor^®^ 647 Goat Anti-Mouse IgG (Life Technologies; Carlsbad, California | a-21235), diluted in blocking solution was added and left at room temperature for 1 hour in the dark. The samples were then washed again three times with washing buffer in the dark for 10 intervals. DAPI (EMD Millipore; Billerica, Massachusetts | 90229) was then added and left for 10 in the dark, followed by three washes in washing buffer for 10 min intervals.

Once the above processing was completed, the samples were mounted on coverslips with Prolong® (Life Technologies; Carlsbad, California | P36934) and kept in the dark at 4 *°*C. Vinculin images were acquired through a FluoView™ 300 Confocal Microscope (Olympus, USA).

### 3.6 Substrate stiffness

Substrate stiffness for the PDMS samples was obtained from the force (*N*) and displacement measurements (mm) from tensile tests performed with an ESM301 Motorized Test Stand (Mark-10; Long Island, New York). Each of the samples (different PDMS ratios) was mounted and subjected to normal tensile testing and subsequently the modulus of elasticity was acquired for each substrate. Five samples were tested for each PDMS ratio to obtain an average of the modulus of elasticity.

### 3.7 Image processing: focal adhesions and vinculin

A criterion was developed for the appropriate processing of the images after considering various scenarios and variables that could influence the results. ImageJ® was selected as the image processing software due to its accessibility and reliability. The criterion is as follows:

○ Previous studies have reported that a cell׳s developmental stage might influence the results obtained through image analysis [1], [2]. To control the developmental stage of the cells and maintain the size of the cells to be analyzed within the average cell size observed, cells had to be spread over an area within the range of 2000 μm^2^ to 5500 μm^2^ and where a single cell can be observed with facility. Although it served as a screening criterion, cell size is not the best indicator of the developmental stage and as a result not all the cells that were analyzed were necessarily at the same stage.
○ Images were obtained far from the edges of the substrate in order to minimize the impact of the cells that detached during the cutting of the substrate for analysis.
○ The threshold method used with ImageJ had to be similar for all the substrates. However, even within the same substrate slight variations were made in order to maximize the number of focal adhesions captured by the software. The image analysis primarily focused on focal adhesions near the cell׳s boundary since these were significantly more abundant compared to focal adhesion points elsewhere in the cell. In most cases, the vinculin cloud prevented us from “seeing” the focal adhesions underneath this cloud (this problem was only present in the cells subjected to cyclic stimulation). Secondly, the “cloud” made it impossible to adequately filter focal adhesions. Analyzing a “clouded” image, required us to lower the “intensity” of the filter resulting in possibly a slight size reduction of the focal adhesions. Hence, a decision was made to remove the vinculin “cloud” and optimize the focal adhesions. The vinculin surrounding the nucleus was manually removed from the images taken in order to focus on the focal adhesions present near the cell’s boundary; for this purpose a filter for a minimum particle size of 0.1 μm^2^ was used. The 0.1 μm^2^ particle size threshold was selected based on trial and error in maintaining a high degree of similarity in the images processed by ImageJ in reflecting the number and size of the focal adhesion points that could be visually appreciated from the confocal image before and after the removal of the signal from the cloud. This 0.1 μm^2^ was then used for all other measurements. Removal of the inactive vinculin around the nucleus allowed the software to adequately quantify the focal adhesions to not allow the noise originating from the vinculin to be misinterpreted as focal adhesions. Subsequently the image without the inactive vinculin around the nucleus removed was analyzed at the threshold used for the quantification of focal adhesions in order to quantify the amount of vinculin present in the sample.
○ Images acquired after the analysis were verified. During this step any other background noise that did not belong to the cell under analysis was removed.

### 3.8 Statistical analysis

Twenty cells were evaluated from each particular substrate. Images taken at a magnification of 60× were evaluated using ImageJ in order to quantify the number of focal adhesions, the area of each focal adhesion and total amount of vinculin present on each sample. These values were then divided by their respective cell areas in order to normalize the data and partially remove cell size as a variable. In addition 10 images of the nuclei on each substrate were taken at a lower magnification of 20*×* and similarly processed through ImageJ^®^ in an attempt to quantify the average number of cells. The data from each test is presented as the mean value±standard error (*SE*). A factorial analysis of variance (*two-way ANOVA*) was performed on the data in order to determine the degree to which the loading condition (*cyclic/static*) and the substrate stiffness (*PDMS 5:1/10:1/15:1*) significantly interacted. Subsequently a least significant difference (*LSD*) Fisher test was performed on the data to establish if a significant difference existed between the loading condition and the substrate stiffness. InfoStat® was used for the statistical analysis of the data obtained from the images.

## 4 Data

Mean values for number and area of the focal adhesion points of MC3T3-E1 cells on the PDMS substrates, amount of vinculin based on analysis of images from immunofluorescent labeling for each PDMS substrate of varying stiffness and loading (cyclic or static) condition were obtained based on two experimental runs. Substrate stiffness, expressed as the modulus of elasticity, was determined for the PDMS substrates used in the culture chambers through standard mechanical testing. The values presented are mean values obtained from five different measurements using linear regression on their respective engineering stress-strain curves. Mean and standard errors, for each substrate׳s modulus of elasticity, *“E”*, are as follows: 2.04±0.06 MPa for PDMS 5, 1.70±0.05 MPa for PDMS 10 and 1.22±0.05 MPa for PDMS 15.

### 4.1 Vinculin immunofluorescence Imaging

A feasible alternative for the identification of focal adhesions is through immunofluorescence labeling of vinculin, since it serves as the stabilizing protein in focal adhesions. The quantity of vinculin relates directly to a cell׳s physiology and behavior on a particular substrate [2]. The experimental data presented in this section are mean values obtained from measurements normalized with respect to the corresponding cell area. Again, data was processed using a factorial analysis and Fisher׳s LSD test for *p<*0.05 in order to determine if the differences observed between the cyclic and static experiments were significant. Both active and inactive vinculin were labeled using this immunofluorescence technique. Inactive vinculin refers to the vinculin that is not located at the focal adhesion sites [3]. Focal Adhesion Area/Vinculin Area was based on the ratio of focal adhesion area to total vinculin area (focal adhesions+vinculin cloud). Table 1 provides relevant data.

**Fig. 1:**
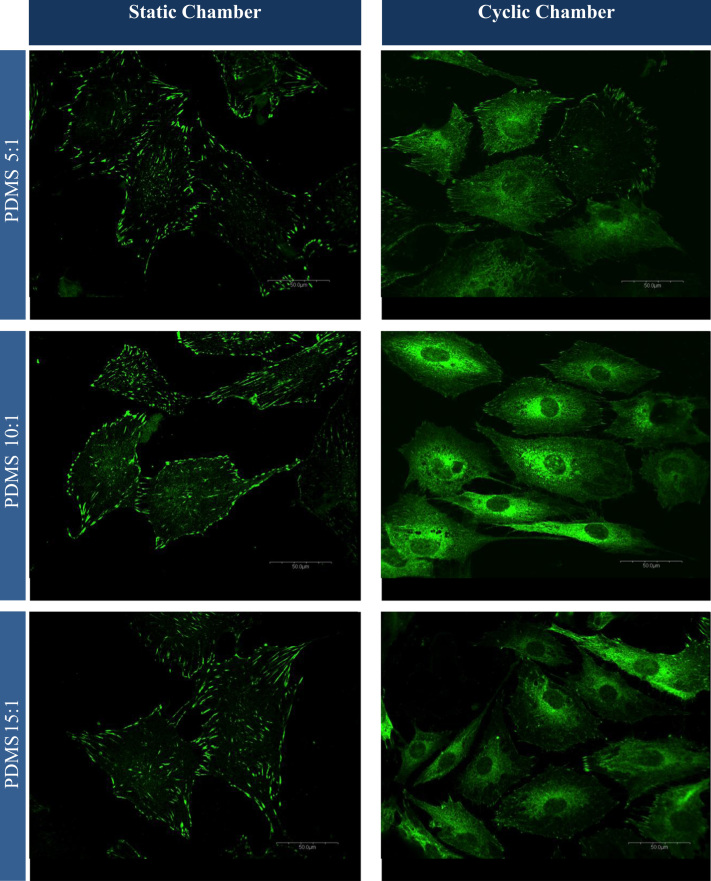
Vinculin expression of MC3T3-E1 cells after cyclic and static loading conditions cultured on PDMS at ratios of 5:1, 10:1 and 15:1 under static conditions (left) and 1% cyclic strain at 0.05 Hz (right). The principal strain axis is parallel to the scale. Each column shows the confocal images of the vinculin expression at a magnification of 60×.

Immunofluorescence images of active vinculin expressed as focal adhesion points and inactive vinculin as a “cloud” is shown in Fig. 1.

## Conflicts of interest

None.

## Figures and Tables

**Table 1:** Focal adhesion characteristics and vinculin expression data.

**Parameters**	**PDMS 5**		**PDMS 10**		**PDMS 15**	
***Cyclic***	***Static***	***Cyclic***	***Static***	***Cyclic***	***Static***
**Density of Focal Adhesions (1**/**μm**^**2**^**)**	0.037	0.053	0.044	0.065	0.037	0.048
**Focal Adhesion Area**	3.45%	7.64%	3.42%	7.34%	3.88%	5.94%
**Vinculin Area**	19.87%	7.79%	23.18%	7.39%	20.53%	5.94%
**Inactive Vinculin Area**	16.42%	0.15%	19.76%	0.05%	16.66%	0%
**Focal Adhesion Area/Vinculin Area**	17.36%	98.07%	14.75%	99.32%	18.90%	100%
